# Validation of a Machine Learning Expert Supporting System, ImmunoGenius, Using Immunohistochemistry Results of 3000 Patients with Lymphoid Neoplasms

**DOI:** 10.3390/diagnostics13071308

**Published:** 2023-03-31

**Authors:** Jamshid Abdul-Ghafar, Kyung Jin Seo, Hye-Ra Jung, Gyeongsin Park, Seung-Sook Lee, Yosep Chong

**Affiliations:** 1 Department of Hospital Pathology, College of Medicine, The Catholic University of Korea, Seoul 06591, Republic of Korea; 2 Department of Pathology, Keimyung University, Daegu 42601, Republic of Korea; 3 Department of Pathology, Korea Institute of Radiological and Medical Sciences, Seoul 01812, Republic of Korea

**Keywords:** database, expert supporting system, machine learning, immunohistochemistry, probabilistic decision tree

## Abstract

(1) Background: Differential diagnosis using immunohistochemistry (IHC) panels is a crucial step in the pathological diagnosis of hematolymphoid neoplasms. In this study, we evaluated the prediction accuracy of the ImmunoGenius software using nationwide data to validate its clinical utility. (2) Methods: We collected pathologically confirmed lymphoid neoplasms and their corresponding IHC results from 25 major university hospitals in Korea between 2015 and 2016. We tested ImmunoGenius using these real IHC panel data and compared the precision hit rate with previously reported diagnoses. (3) Results: We enrolled 3052 cases of lymphoid neoplasms with an average of 8.3 IHC results. The precision hit rate was 84.5% for these cases, whereas it was 95.0% for 984 in-house cases. (4) Discussion: ImmunoGenius showed excellent results in most B-cell lymphomas and generally showed equivalent performance in T-cell lymphomas. The primary reasons for inaccurate precision were atypical IHC profiles of certain cases, lack of disease-specific markers, and overlapping IHC profiles of similar diseases. We verified that the machine-learning algorithm could be applied for diagnosis precision with a generally acceptable hit rate in a nationwide dataset. Clinical and histological features should also be taken into account for the proper use of this system in the decision-making process.

## 1. Introduction

Immunohistochemical staining (IHC) is a unique antigen–antibody reaction method that is used for pathological diagnosis by staining tissue sections [1,2,3,4,5,6]. It is an essential process in pathologic diagnosis and is often very challenging owing to exponentially increasing IHC data and complex cases of hematolymphoid diseases [1,2,3,5,6,7,8]. Hematolymphoid neoplasms are mainly classified as B-, T-, and NK/T cells and histiocytic neoplasms according to IHC profiles, and each of these lymphomas can be divided into many subtypes arising from every developmental stage of mature and immature lymphocytes, which may need different IHC marker profiles [1,4,6,9]. Therefore, the accurate pathological diagnosis of each subtype mainly depends on the appropriate selection of IHC panels, knowledge of the pathologist, and interpretation of the IHC results, which could easily be biased by individual pathologist experience [1,5,10]. Many new IHC antibodies for different hematolymphoid neoplasms are introduced annually, and over a hundred thousand studies applying IHC have been published since 2000 just in the human brain, which makes it difficult to memorize all the newly developed antibodies and understand the expression characteristics of various tumors [11,12,13,14,15]. In addition, recent developments in digital pathology require a proper reference database of IHC tests to combine medical knowledge with individual medical problems [16]. Attempts have been made to address this problem by adopting an algorithmic approach or using standardized IHC panels for specific and differential diagnosis [8,14,17]. However, the clinical situation of each case, particularly in hematolymphoid neoplasms, is unique and case-sensitive, and generalized application of a particular IHC panel or specific algorithm is not easy and can be time-consuming and labor-intensive.

To support this qualitative analysis, a machine-learning based expert supporting system, ImmunoGenius, was recently developed as a mobile application (iOS and Android) and showed a generally acceptable hit rate of 95% in the diagnosis of lymphoid neoplasms [2]. However, a high hit rate was mostly observed in B-cell lymphomas, including diffuse large B-cell (DLBCL), follicular, mucosa-associated lymphoid tissue (MALT), and Burkitt lymphomas, with zero errors. An incorrect predictive diagnosis was observed in plasmablastic, mantle cell (MCL), extranodal NK/T-cell, and nasal type lymphomas, along with T-lymphoblastic leukemia/lymphoma. In Hodgkin lymphomas, the error rate was 50%. All these errors were associated with atypical IHC profiles, lack of site- and disease-specific markers, overlapping IHC profiles between disease entities, and mainly due to lack of external validation with a large number of cases.

To reduce biases in IHC selection and predict differential diagnoses more accurately by pathologists using ImmunoGenius, we evaluated the prediction accuracy of this software using nationwide data from 25 university hospitals in Korea to externally validate the clinical utility of this application.

## 2. Materials and Methods

This study was approved by the Institutional Review Board of The Catholic University of Korea College of Medicine (SC17RCDI0074). The requirement for informed consent was waived with the permission of the Institutional Review Board.

### 2.1. Machine-Learning Expert Supporting System, ImmunoGenius

**Figure 1:**
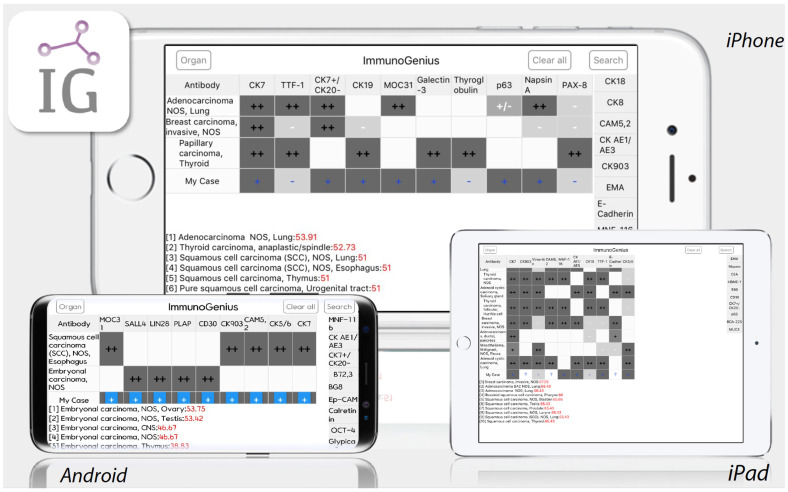
Machine-learning based mobile IHC interpretation supporting software, “ImmunoGenius”.

ImmunoGenius is a reactive native mobile application developed with NoSQL for iOS and Android platforms in 2018, which can be universally used in iPhones, iPads, Android phones, and tablet devices (Figure 1).

**Figure 2:**
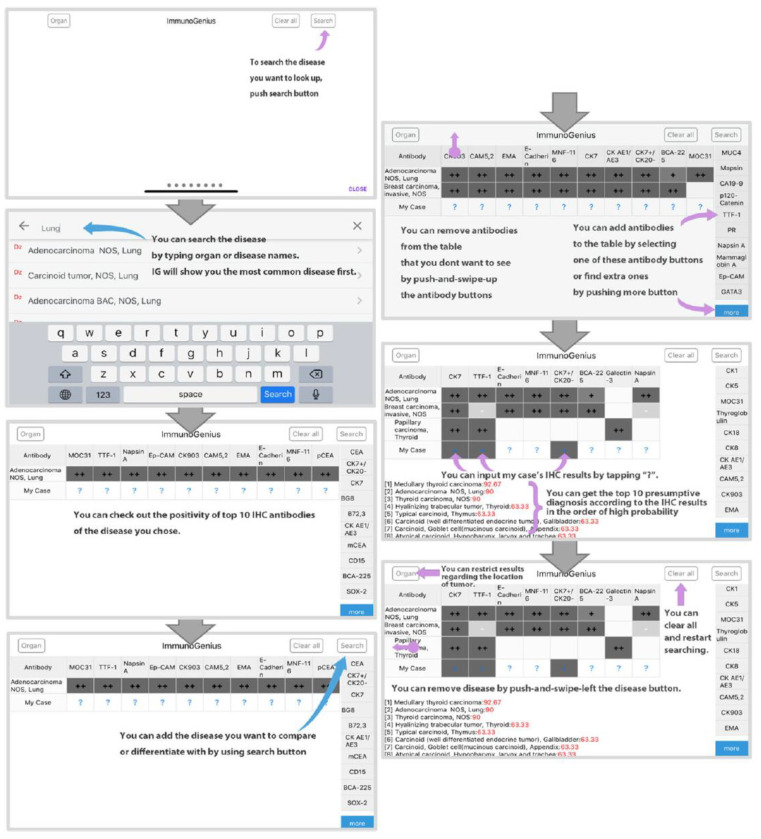
Exemplary flowchart for ImmunoGenius.

It was designed to search and select targeted differential diagnoses and generate a 2 × 2 table with disease names in the left column and IHC antibody names in the first row (Figure 2 and Supplementary Video S1). Representative IHC profiles appear in the corresponding cells as ++ for 75–100% positivity, + for 50–74%, +/− for 30–49%, −/+ for 10–29%, and − for 0–9% with graded shades. Users can compare IHC profiles between selected diseases and add or remove rows (diseases) or columns (IHC antibodies) to customize the table. Additional IHC profiles can be added using buttons on the right side. Once the user inputs the IHC results for their case, the 10 most probable diagnoses as calculated by the diagnosis precision algorithm appear below, along with the estimated probability in percentage (red numbers) (Figure 2 and Supplementary Video S1). The key mechanism of this mobile application is based on Bayes’ theorem and the probabilistic decision-tree-like nature of IHC results (Supplementary Figure S1). The database of the IHC expression profile of approximately 600 antibodies in about 5000 neoplasms was built based on knowledge from major textbooks and literature, including the World Health Organization (WHO) Classification of Tumors series (IARC, Lyon, France). The IHC profiles of over 150 types of lymphoid neoplasms, according to the WHO classification, were also included.

In a previous developmental validation study, IHC profile data and diagnoses originally made by pathologists were compared with the top 10 predictive diagnoses produced by the algorithm. IHC profile data of 994 patients with lymphoma were obtained from the archives of two independent university hospitals, Yeouido and Seoul St. Mary’s Hospital, College of Medicine, The Catholic University of Korea, from 2010 to 2017. Approximately 80% of lymphoma cases at Seoul St. Mary’s Hospital were referred from various institutes in Korea. The retrieved data were divided 6:4 for training and validation purposes. Cases with inconclusive diagnoses or inadequate IHC profiles (fewer than three antibodies, inconclusive results, and absence of markers for tumor origins, but only prognostic or therapeutic markers, such as epidermal growth factor receptor or p53) were excluded. The diagnosis precision hit rate was determined by including the original diagnosis in the top 10 predictive diagnoses drawn by the algorithm. An inclusive hit rate was when there was no significant difference in the IHC profile between the original and predictive diagnoses, and the only difference was in location if the two diagnoses shared the same origin of cells (e.g., nodal vs. extranodal marginal zone lymphoma). The algorithm was validated by comparing the hit rates of the training and validation data for lymphomas.

### 2.2. External Validation Using Nationwide IHC Dataset of Lymphoid Neoplasms

To expand the diagnostic utility and validity, we tested a larger nationwide dataset of IHC profiles collected from 25 university hospitals with the help of the Korean Study Group of Hematopathology. IHC profile data for 3722 patients with lymphoma diagnosed between 2015 and 2016 were retrieved from the archives of 25 independent university hospitals, which represent the national population (Table 1).

This sample size is also reported in the previous publication of our research group by Jung et al. The sample size used in Jung et al.’s included a total of 7737 patients, and the distribution of diagnosis according to anatomical site was evaluated in 7689 patients [18]. Cases with ambiguous diagnoses, incomplete IHC data, or duplicated cases due to hospital transfer were excluded in the present study and only IHC data from 3722 patients were used. The common anatomic location for the acquired sample used in the Jung et al. study was as follows: the extranodal site commonly involved in certain anatomical regions such as the gastrointestinal tract (35.7%), bone and soft tissue (10%), Waldeyer’s ring (7.1%), and the central nervous system (7.0%). The gastrointestinal tract and eye were most commonly affected by MALT lymphoma, while ENKTL lymphoma was more prevalent in the nasal region. Furthermore, information regarding the source of the acquired specimen can be found in Jung et al.’s publication from our research group [18].

All patient data related to identification, except for the original diagnosis and IHC results, were blinded before data processing. The presumptive diagnostic accuracy was determined following the same method as the training and validation process by the inclusion of the original diagnosis in the top 10 presumptive diagnoses drawn by the application.

### 2.3. Statistical Analysis

Time and computational complexity were evaluated by testing the mobile application. The chi-square test was used to compare the accuracy of the original and presumptive diagnoses. The statistical analysis was performed using a web-based statistical analysis by Web-R (http://web-r.org (accessed on 8 February 2023)).

## 3. Results

### 3.1. External Validation Data Characteristics

The original diagnoses from the external validation data are presented in Table 2. A total of 2993 lymphoma cases were retrieved for external validation. In the external validation data, diffuse large B-cell lymphoma, not otherwise specified (DLBCL, NOS) was the most common, with 1239 cases (41.3%), and the second most common was extranodal marginal zone lymphoma of mucosa associated lymphoid tissue (MALT), with 436 cases (14.5%). Follicular lymphoma, with 203 cases (6.7%), was the third most common in the external validation dataset. In the external validation data, more than 3% of cases were extranodal NK/T-cell lymphoma (nasal type), while angioimmunoblastic T-cell lymphoma, peripheral T-cell lymphoma, NOS were 121 (4.0%), 110 (3.6%), and 103 (3.4%), respectively. In addition, the remaining lymphoma cases in the external validation data were less than 3%.

### 3.2. External Validation Results

In the external validation data, the hit rate for the predictive diagnosis (top 10) was 90.7% (Table 3). Detailed results of the discordant cases between the original training and external validation data of the presumptive diagnoses are presented in Table 2. The hit rate of the presumptive diagnosis in the first, second, and third most common type was excellent, with few error cases in DLBCL, NOS (4.5%, 56/1239), MALT lymphoma (14.4%, 63/436), and follicular lymphoma (5.4%, 11/203), respectively. The hit rate of the presumptive diagnosis was also excellent, with few error cases in angioimmunoblastic T-cell lymphoma (10.9%, 12/110), primary DLBCL of the CNS (5.0%, 3/60), chronic lymphocytic leukemia/small lymphocytic lymphoma (1.8%, 1/57), and mantle cell lymphoma (1.4%, 1/73). It showed generally good performance in most B-cell lymphomas except for B-cell lymphoma that was unclassifiable, with features intermediate between DLBCL and Burkitt lymphoma (10 errors out of 13 cases, 76.9%) and primary cutaneous follicle center lymphoma (two errors out of four cases, 50.0%). In T-cell lymphomas, the hit rate showed generally equivalent performance to B-cell lymphomas, except for T lymphoblastic leukemia/lymphoma (only 1 error in 44 cases, 2.3%). In enteropathy-associated T-cell lymphoma (types 1 and 2), primary cutaneous CD4 + small/medium T-cell lymphoma, peripheral T-cell lymphoma, NOS, and ALCL, ALK-negative, the error rates were 38.9, 75.0, 12.6, and 16.2%, respectively. The error rates were 66.7% in nodular lymphocyte-predominant Hodgkin lymphoma, 17.3% in nodular sclerosis classical Hodgkin lymphoma, and 18.8% in classical Hodgkin lymphoma, NOS. In the external validation of the presumptive diagnosis, the hit rate was 100% (no error) in primary effusion lymphoma, Burkitt lymphoma, and splenic B-cell lymphoma/leukemia unclassifiable cases (0/1, 0/42, and 0/1, respectively). The hit rate of presumptive diagnoses showed an almost 100% error rate in cases where training data were not available, such as primary cutaneous gamma-delta T-cell lymphoma, hepatosplenic T-cell lymphoma, and splenic B-cell lymphoma/leukemia, and thus, unclassifiable.

### 3.3. The Presumptive Error Rates between Training, Validation, External Validation Datasets, and Performance in Computational Time

The error rates of presumptive diagnosis were 5.3, 4.3, and 9.3% in the training, validation, and external validation datasets, respectively. The overall accuracy rate was 91.8% for lymphomas (Table 3). The application also exhibited acceptable hit rates of 94.7 and 95.7% in the training and validation datasets, respectively (Table 3). The hit rates between training, validation, and external validation differ significantly.

Providing an analysis of computational time is crucial when evaluating machine learning or deep learning algorithms. Comparing the time consumed during the diagnosis process with and without the application is challenging both in terms of time and user experience. This ImmunoGenius algorithm requires IHC data as input. The algorithm operates on a native database of less than 5 MB and uses Bayesian theorem to generate probabilistic diagnostic results that can be updated in real time as new IHC results are input or changed.

## 4. Discussion

We externally validated the previously verified calculation of the probability of lymphoma using the IHC results from a probabilistic decision tree and corresponding mobile application, ImmunoGenius. IHC profile data from 3722 cases were collected for external validation. IHC profiles of these cases were collected from 25 university hospitals nationwide with the help of the Korean Study Group of Hematopathology. The presumptive diagnosis drawn by the probabilistic decision tree algorithm was convincing, with an accuracy of 91.8% for lymphomas.

The accuracy of the presumptive diagnosis algorithm in the validation data was excellent for most B-cell lymphomas, including DLBCL, follicular lymphoma, CLL/SLL, MALT lymphoma, and Burkitt lymphoma with zero error, which consisted majority of all lymphoma cases (approximately two-thirds) [2]. However, the external validation data, which included approximately 3000 cases from 25 university hospitals, revealed low errors in the same lymphomas, including DLBCL (4.5%), follicular lymphoma (5.4%), CLL/SLL (1.8%), and MALT lymphoma (14.4%). The percentage of errors in the external validation data are very small and are due to the large number of lymphoma cases in the external validation data compared to the training and validation data. The error for Burkitt’s lymphoma was zero. One case of MCL in the previous validation showed an incorrect presumptive diagnosis, which was an atypical case of a cyclinD1-negative MCL (IHC results: CD20 +, Bcl-2 +, CD3 −, CD10 −, Bcl-6 −, CD23 −, MUM1 −, and p53 −). The external validation data also showed an incorrect presumptive diagnosis for a case of MCL with unusual IHC findings (IHC results: CD3 +, CD8 +, CD56 +, CD4 +, CD20 −, CD21 −, and EBV −). Both cases were confirmed by fluorescence in situ hybridization with CCND1/IGH translocation. In plasmablastic lymphomas, the training data showed incorrect presumptive diagnosis in all cases (3/3 cases), while in the external validation, the error rate decreased from 100 to 63.6% (7/11 cases). The IHC profile of plasmablastic lymphoma is relatively similar to that of other plasma cell neoplasms, large B-cell lymphomas, and MALT lymphoma, in which CD30, CD38, CD138, and CD79a are positive; however, CD20 is often negative [2,3,19,20]. All three retrieved cases of plasmablastic lymphoma in the training data showed positive CD20, while the four correct cases of plasmablastic lymphoma in the external validation data were negative for CD20. All the incorrectly diagnosed cases were presumed to be either plasma cell neoplasms, such as multiple myeloma and solitary plasmacytoma, or B cell neoplasms, such as DLBCL, anaplastic variant (CD30 positive), and extranodal MALT lymphoma with plasmacytoid differentiation. We believe that the main reason for the error in presumptive diagnosis could be the lack of a disease-specific marker and IHC profile overlapping with similar diseases.

In primary cutaneous follicle center lymphoma, both validation (1/2) and external validation (2/4) datasets showed a 50% error in the presumptive diagnosis. The main reason for this inaccurate presumption could be due to the similarity of the IHC profile to follicular lymphoma. The ImmunoGenius algorithm does not consider clinicopathologic information, such as tumor location and skin versus lymph node, which could explain the incoherence in this case.

The algorithm showed heterogeneous performance in T-cell lymphomas compared to B-cell lymphomas, except for ALCL and ALK-positive, which showed no error in presumptive diagnosis in all datasets (training, validation, and external validation). T lymphoblastic leukemia/lymphoma and extranodal NK/T-cell lymphoma, nasal type revealed zero errors in training and validation data; however, due to the large number of cases, a small number of errors were found in the external validation data. A relatively accurate presumptive diagnosis in T lymphoblastic leukemia/lymphoma and extranodal NK/T-cell lymphoma, nasal type, could be due to the presence of disease-specific markers, such as TdT, CD56, and EBER. However, external validation data for other T-cell lymphomas, such as adult T-cell leukemia/lymphoma, enteropathy-associated T-cell lymphoma (types 1 and 2), peripheral T-cell lymphoma, NOS, ALCL, ALK-negative, primary cutaneous gamma-delta T-cell lymphoma, and angioimmunoblastic T-cell lymphoma, showed a high range of presumptive diagnostic error rates from 10.9 to 100.0%. Adult T-cell leukemia/lymphoma, which showed 100 and 50% error rates in the training and external validation datasets, respectively, had similar IHC profiles to peripheral T-cell lymphoma, NOS, without disease-specific markers, but only distinctive clinicopathologic features [2,19,20]. Similarly, enteropathy-associated T-cell lymphomas also have no specific diagnostic IHC markers but distinctive clinicopathologic findings and often share IHC profiles with peripheral T-cell lymphomas. Moreover, ALCL, ALK-negative lymphoma shows anaplastic morphology with negative ALK, which can share IHC profiles with peripheral T-cell lymphoma, NOS, Hodgkin lymphomas, and even with ALCL, ALK-positive type [2,19,20]. In angioimmunoblastic T-cell lymphoma, which revealed a lower presumptive error in external validation data (10.9%) compared to training (50%) and validation data (42.8%), programmed death-1 (PD-1) can be considered a specific diagnostic marker [21]. However, many other lymphomas also express PD-1 at a variable rate, and its positivity is often interpreted based on different histologic characteristic features [3,10,19]. A different category of nodal and extranodal mature T-cell lymphomas is peripheral T-cell lymphoma, NOS, which does not correspond to any of the entities clearly defined [20]. Therefore, the IHC profile of this lymphoma covers a wide variety of different expressions and is often confused with that of other T-cell lymphoma entities. In summary, T-cell lymphomas often show similar IHC profiles and have no disease-specific IHC markers but can be differentially diagnosed based on clinicopathologic findings with or without IHC profiles.

In Hodgkin lymphomas, the diagnostic error rates were 66.7% in nodular lymphocyte-predominant Hodgkin lymphoma (4/6), 18.8% in classical Hodgkin lymphoma, NOS (3/16), 17.3% in the nodular sclerosis subtype (12/69), and 14.2% in the mixed cellularity subtype (9/63), which were relatively similar to the findings from the training and validation data. The nodular lymphocyte-predominant subtype of Hodgkin lymphoma shares an IHC profile with T-cell/histiocyte-rich DLBCL and ALCL, ALK-negative, and similar clinicopathologic features. Classical Hodgkin lymphoma and its subtypes share IHC profiles with peripheral T-cell lymphoma, NOS. The differential diagnosis between these two entities is not possible based only on the IHC profiles, especially if the specific marker of Hodgkin lymphoma, CD15, is negative. Therefore, integrated and comprehensive diagnosis, including clinicopathologic findings, in addition to the possible diagnosis using IHC, is essential.

The current study supports our previous work and validates the feasibility and clinical utility of presumptive diagnosis algorithm with the corresponding mobile application ImmunoGenius, using IHC profiles in the differential diagnosis of lymphomas. The overall accuracy rate of this machine-learning algorithm was 91.8% for lymphomas using training, validation, and external validation data. The main reasons for the errors were atypical IHC profiles, a lack of site-specific, disease-specific markers, overlapping IHC profiles between disease entities, and mixed/combined tumors. Although this system can be a useful tool for pathologists to make better decisions during the process of pathological diagnosis by having a wide range of IHC profiles relevant to efficient and accurate differential diagnosis, integrated interpretation with contextual information, such as clinical, radiological, and pathological findings is highly recommended. Supportive use of this application is more desirable. Further work to develop an application of artificial neural network algorithms to optimize the disease, organ incidence, and antibody weight is recommended in the future.

### 4.1. Contribution of the Study

The major contribution of this multi-institutional study is the validation of a machine-learning algorithm on a larger scale dataset of 3977 cases from 25 independent university hospitals in Korea, which represents a nationwide clinical data and could reflect the real-world data of the lymphoma cohort of Korea. The data collected between 2015 and 2016 represents the national population of Korea. The results show an overall hit rate of 91.8% for the algorithm, demonstrating its potential to assist pathologists in making accurate diagnoses. With such a large amount of data, this study provides robust evidence for the effectiveness of this machine-learning algorithm, which could significantly improve diagnostic accuracy for lymphoma.

### 4.2. Limitations of the Study

DLBCL, NOS represent a significant portion of the data sets used in this study. The ImmunoGenius system shows a relatively high accuracy in classifying DLBCL (4.5% error rate). We presume that the highest proportion of the IHC data of DLBCL, NOS in the training (216/602, 35.8%) and validation (145/392, 40.0%) could contribute to the system’s high accuracy in classifying this entity, whereas some of other entities account relatively low amount of both the training set (TS) and the validation set (VS), such as nodal marginal zone lymphoma (4/602, 0.6% of TS; 5/392, 1.2% of VS), plasma cell myeloma (4/602, 0.6% of TS; 0/392, 0.0% of VS), follicular lymphoma (4/602, 0.6% of TS; 22/392, 5.6% of VS), and MALT-lymphoma (78/602, 13.0% of TS; 74/392, 18.8% of VS). The lower amounts of IHC data of some entities for TS and VS, which could result in a higher level of error in external validation. However, the incidence rates of lymphoma subtypes in this study represent the real-world data of the Korean population, as the samples have been collected from the clinical data of more than 25 university hospitals for two years. The high error rate is also due to unusual IHC profiles, the absence of markers specific to the site and disease, similarities in IHC profiles between different diseases, and the presence of mixed or combined tumors. Another limitation of the study is that we validated ImmunoGenius with the only data from the Korean ethnic population.

The machine learning algorithms require a large and diverse training dataset. For rare subtypes of lymphoma, it can be challenging to obtain sufficient data to train the algorithm accurately. However, past research has produced AI models that demonstrate high accuracy in lymphoma classification but the sample size of these studies is small [22,23]. Additionally, another study developed an AI model capable of distinguishing DBCL from non-lymphoma samples [24]. Furthermore, there is currently a lack of AI/machine learning algorithms tested on a large multiethnic population data in order to produce a generalizable outcome. As the AI field continues to expand, further work is required with high-quality multi-ethnic dataset.

### 4.3. Future Direction

**Figure 3:**
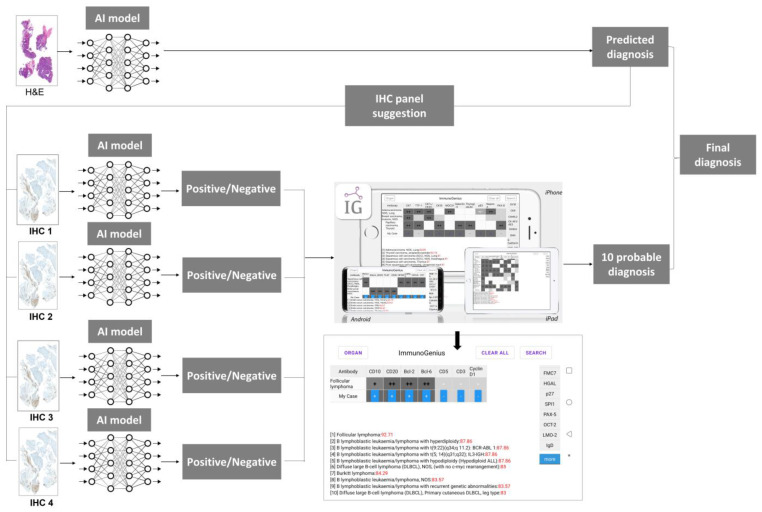
A schematic diagram showing the integrated H&E, IHC AI model with ImmunoGenius.

A schematic diagram of the integrated Hematoxylin and eosin (H&E), IHC AI model with ImmunoGenius is shown in Figure 3. With the growth of the AI field, numerous AI tools are being developed to predict lymph node metastasis, diagnose hematological disorders, and detect breast cancer and be applicable in many other areas of science [25,26,27,28,29,30]. Previous research studies have shown that AI models demonstrate good performance in various aspects of tumor detection, classification, gland segmentation, and grading in many types of cancers [31,32,33,34,35]. However, the development of AI models for the analysis of IHC is ongoing, and a good AI model for accurate classification of IHC is yet to be developed. Once a good AI model for IHC is available, it can be integrated with our ImmunoGenius tool. Based on the results of AI models that predict diagnosis on H&E images, a panel of IHC can be suggested for further differential diagnosis. The integration of AI models for cancer diagnosis, IHC panel suggestion, and IHC interpretation, along with ImmunoGenius, can potentially revolutionize the field of cancer diagnosis and treatment. By analyzing vast amounts of data, these models can help identify IHC markers, and ImmunoGenius can make predictions with this input data. Employing these integrated models can help in the accurate diagnosis of cancer and in reducing intra-observer variability. The AI-based model for diagnosis not only helps in reducing the time and cost of diagnosis but also improves accuracy and reliability. Overall, in the future, the integration of AI models for cancer diagnosis from H&E slides, IHC interpretation AI models, and our ImmunoGenius holds significant promise for improving cancer diagnosis and treatment, and ongoing research in this field is likely to yield even more benefits in the future.

## 5. Conclusions

The mobile application ImmunoGenius produced acceptable diagnostic hit rates for all data, including the training, validation, and external validation datasets. The overall hit rate of this machine-learning algorithm was 91.8%, which was slightly lower than that of the previous validation data (95.7%). However, the current data represents the entire national population from 25 university hospitals around the country. In differential diagnosis, due to the lack of specific markers of some lymphomas, clinical data, and histological features should be considered to make proper use of this system in the pathologic decision-making process. This system will be useful in assisting pathologists in making precise decisions during diagnosis. Further studies to recommend IHC panels for particularly complex problems regarding differential diagnosis and application of artificial neural network algorithms are needed in the future.

## Figures and Tables

**Table 1:** Immunohistochemistry profile data from 25 university hospitals.

No.	Name of the Institute	No. of Cases
1	Ajou University Hospital	456
2	Asan Medical Center	113
3	Chonnam National University Hwasun Hospital	434
4	Chonnam National University Hospital	146
5	Dong-A University Hospital	117
6	Eulji University Hospital	61
7	Ewha Womans University Mokdong Hospital	83
8	Gyeongsang National University	102
9	Gyeongsang National University Changwon Hospital	35
10	Hallym University Sacred Heart Hospital	93
11	Inje University Haeundae Paik Hospital	117
12	Inje University Sanggye Paik Hospital	49
13	Jeonbuk National University Hospital	206
14	Keimyung University Dongsan Medical Center	137
15	Konkuk University Hospital	58
16	Korea University Guro Hospital	160
17	Kosin University Gospel Hospital	95
18	Nowon Eulji Medical Center, Eulji University	16
19	Presbyterian Medical Center	74
20	Seoul Metropolitan Government-Seoul National University Boramae Medical Center	152
21	Soonchunhyang University Bucheon Hospital	112
22	Soonchunhyang University Seoul Hospital	59
23	The Catholic University of Korea, Seoul St. Mary’s Hospital	591
24	The Catholic University of Korea, Yeouido St. Mary’s Hospital	59
25	Ulsan University Hospital	197
	**Total No. of cases**	**3722**

**Table 2:** Cases with discordant results between the original and predictive diagnoses in training, validation, and external validation data.

Type of Lymphoma	Training Data		Validation Data		External Validation Data	
	Error/No.	%	Error/No.	%	Error/No.	%
B lymphoblastic leukemia/lymphoma	0/8	0	0/5	0	1/20	5.0
Chronic lymphocytic leukemia/small lymphocytic lymphoma	0/20	0	0/11	0	1/57	1.8
Extranodal marginal zone lymphoma of MALT (lymphoma)	0/78	0	0/74	0	63/436	14.4
Nodal marginal zone lymphoma	0/4	0	0/5	0	11/36	30.5
Plasma cell myeloma	0/3	0	0/0	0	4/31	12.9
Follicular lymphoma	0/62	0	0/22	0	11/203	5.4
Mantle cell lymphoma	1/24	4.2	0/19	0	1/73	1.4
DLBCL						
-NOS	0/216	0	0/145	0	56/1239	4.5
-T-cell/histiocyte-rich	0/2	0	0/0	0	0/7	0.0
-Primary DLBCL of the CNS	0/9	0	0/0	0	3/60	5.0
-Associated with chronic inflammation	-	-	-	-	0/2	0.0
-EBV positive DLBCL of elderly	0/1	0	0/0	0	2/29	6.9
-Primary cutaneous DLBCL, leg type	0/0	0	0/1	0	0/3	0.0
Primary mediastinal (thymic) large B-cell lymphoma	0/9	0	0/3	0	5/29	17.2
Plasmablastic lymphoma	3/3	100	0/0	0	7/11	63.6
Primary effusion lymphoma	0/1	0	0/0	0	0/1	0.0
Burkitt lymphoma	0/17	0	0/11	0	0/42	0.0
B-cell lymphoma, unclassifiable, with features intermediate between DLBCL and Burkitt lymphoma	0/3	0	0/0	0	10/13	76.9
Primary cutaneous follicle center lymphoma	0/0	0	1/2	50	2/4	50.0
T lymphoblastic leukemia/lymphoma	0/17	0	0/7	0	1/44	2.3
Extranodal NK/T-cell lymphoma. nasal type	0/25	0	0/15	0	8/121	6.6
Adult T-cell leukemia/ lymphoma	1/1	100	0/0	0	1/2	50.0
Enteropathy-associated T-cell lymphoma						
-Type 1	-	-	-	-	2/2	100.0
-Type 2	-	-	-	-	5/16	31.3
Mycosis fungoides	0/0	0	0/3	0	3/22	13.6
Primary cutaneous (CD30-positive T-cell) ALCL	0/1	0	0/0	0	6/12	50.0
Primary cutaneous gamma-delta T-cell lymphoma	-	-	-	-	1/1	100.0
Subcutaneous panniculitis-like T-cell lymphoma	0/0	0	0/1	0	2/6	33.3
Peripheral T-cell lymphoma, NOS	9/23	34.7	4.12	33.3	13/103	12.6
Angioimmunoblastic T-cell lymphoma	8/16	50	3/7	42.8	12/110	10.9
ALCL, ALK-positive	0/5	0	0/2	0	0/33	0.0
ALCL, ALK-negative	3/9	33.3	2/6	33.3	6/37	16.2
Nodular lymphocyte-predominant Hodgkin lymphoma	0/0	0	1/2	50	4/6	66.7
Classical Hodgkin lymphoma, NOS	1/8	12.5	2/11	18.2	3/16	18.8
Nodular sclerosis classical Hodgkin lymphoma	3/21	14.3	1/5	20	12/69	17.3
Mixed cellularity classical Hodgkin lymphoma	0/7	0	0/8	0	9/63	14.2
Hepatosplenic T-cell lymphoma	-	-	-	-	1/1	100.0
Lymphocyte rich Classical HL	-	-	-	-	2/15	13.3
Lymphomatoid granulomatosis	-	-	-	-	3/4	75.0
Lymphoplasmacytic lymphoma	-	-	-	-	2/6	33.3
Primary cutaneous CD4 positive small/mediumT-cell lymphoma	-	-	-	-	3/4	75.0
Splenic B-cell lymphoma/leukemia, unclassifiable	-	-	-	-	0/1	0.0
Splenic B-cell marginal zone lymphoma	-	-	-	-	1/1	100.0
Systemic EBV+ T-cell lymphoproliferative disease of childhood	-	-	-	-	1/2	50.0
**Total**	**32/602**	**5.3**	**17/392**	**4.3**	**278/2993**	**9.2**

**Table 3:** A comparison of precision error rates for training, validation, and external validation datasets of lymphoma cases.

Precision Diagnosis	Training Data (%)	Validation Data (%)	External Validation Data (%)	Total (%)
Accurate results	570 (94.7)	365 (95.7)	2715 (90.7)	3650 (91.8)
Error results	32 (5.3)	17 (4.3)	278 (9.3)	327 (8.2)
Total	602 (100)	382 (100)	2993 (100)	3977 (100)

## Data Availability

The datasets used and/or analyzed during the current study are available from the corresponding author upon reasonable request.

## Supplementary Materials

The following supporting information can be downloaded at: https://www.mdpi.com/article/10.3390/diagnostics13071308/s1, Figure S1: (A) Probabilistic decision tree for a machine-learning algorithm in diagnostic tests and diseases. (B) Prior and post probability based on Bayes’ Theorem; Video S1: Exemplary video for ImmunoGenius.

## Patents

Copyright of ImmunoGenius, the mobile application developed during this project, is owned by the Catholic University of Korea, Industry-Academic Cooperation Foundation. The content of the software, including the idea, database, user interface, and source code, was protected.
