# Supplementary material for: Validation of a Machine Learning Expert Supporting System, ImmunoGenius, Using Immunohistochemistry Results of 3000 Patients with Lymphoid Neoplasms
Source: Diagnostics (Basel). 2023 Mar 31;13(7):1308. doi: 10.3390/diagnostics13071308 (PMC10093096; doi:10.3390/diagnostics13071308)
Supplement: Supplementary file 1 [file diagnostics-13-01308-s001.zip › diagnostics-2240536-Figure S1.pdf]

A

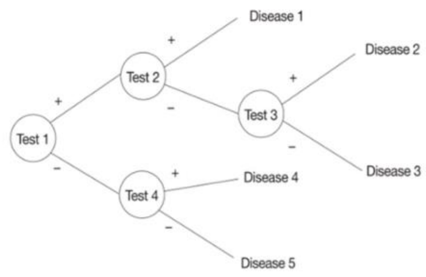

B

	Test A	Text B	Text C	Prior probability		Post probability
Disease 1 (30%)	60%	30%	0%	0.3 ×	0.6 × 0.7 × 0.0	= 0.000
Disease 2 (50%)	20%	80%	10%	0.5 ×	0.2 × 0.2 × 0.1	= 0.002
Disease 3 (20%)	50%	50%	70%	0.2 ×	0.5 × 0.5 × 0.7	= 0.035
Test results	+	−	+	The probability of disease 3 is the highest with the test results		

Figure S1. (**A**) Probabilistic decision tree for a machine-learning algorithm in diagnostic tests and diseases. (**B**) Prior and post probability based on Bayes' Theorem.
